# Allowing caterpillar locomotion on any substrates: towards hybrid soft robots design

**DOI:** 10.1098/rsif.2026.0288

**Published:** 2026-10-21

**Authors:** Mario Argenziano, Arsenio Cutolo, Massimiliano Zingales, Massimiliano Fraldi

**Affiliations:** ^1^ Department of Engineering, University of Palermo, Palermo, Sicily, Italy; ^2^ Department of Structures for Engineering and Architecture, University of Naples Federico II, Napoli, Campania, Italy

**Keywords:** caterpillar locomotion, crawling gait's mechanics, interplay elasticity–adhesion, soft robotics

## Abstract

Soft robotics draws inspiration from nature to develop efficient locomotion strategies. Among natural organisms, caterpillars serve as excellent biomimetic models by exploiting body elasticity and substrate adhesion. Building on previous studies, this work employs a crawling model that represents the body as spring-linked masses, interacting with the environment via threshold-based adhesion and Winkler-like springs. To experimentally further validate this theoretical framework, we analysed the locomotion of *Pieris brassicae* larvae across substrates with diverse morphologies: wood, steel, slate and glass. Our results highlight a strong correlation between substrate roughness and crawling performance. While caterpillars seamlessly adapt their movement to predominant surface patterns, they struggle on smooth surfaces like glass—where microscale asperities are insufficient for proleg grip—confirming that adequate adhesion is crucial to trigger and sustain movement. Driven by the model's insights on these physical constraints, we explored how crawling mechanics could be artificially enhanced. Through proof-of-concept experiments, we equipped larvae with artificial armour to increase body stiffness and additional masses to improve effective friction and adhesion, allowing them to cross non-walkable glass surfaces. Ultimately, these findings validate the model's predictive accuracy, envisioning possible novel design criteria for bioinspired soft crawlers capable of adapting movements in complex habitats.

## 1. Introduction

In recent years, the locomotion strategies of biological organisms have attracted considerable scientific interest owing to their remarkable efficiency and adaptability across diverse and intricate environmental conditions. These mechanisms have become a major source of inspiration in the development of novel bioinspired systems tailored for both engineering and medical applications [1–11].

Within the vast range of locomotion models exhibited by biological systems [12–22], caterpillars stand out owing to their segmented and flexible body structure, making them particularly suitable references for the advancement of soft robotic research [23–25], since their crawling gait demonstrates a highly stable and effective motion modality [15,26–30].

The versatile locomotion of caterpillars across different surfaces [31] is widely governed by several key morphological traits. Covered by a soft cuticle, the caterpillar body is typically divided into 12--14 segments, some of which are equipped with legs or prolegs [1,26,28,29,32,33]. These appendages serve distinct functions: the suction cup-like abdominal prolegs drive locomotion by cyclically attaching to and releasing from the substrate, whereas the thoracic legs are primarily dedicated to sensing and exploring the environment [28,34–38]. In addition, locomotion is supported by elastic longitudinal muscles within the body, which can contract to less than half their resting length and extend to nearly double it [34,39,40]. Literature evidence [28,40] shows that locomotion is driven by a muscle contraction localized at the rear body portion that creates a forward-moving wave, causing the prolegs to sequentially detach and reattach to the surface [28,40]. In a previous work [38], we demonstrated, by integrating mechanical modelling and experimental tests, that this periodic crawling is ruled by the competition between body elasticity and adhesion in caterpillar locomotion. Specifically, laboratory observations confirm that as the contraction wave progresses, it stores enough elastic energy to overcome adhesion, lifting the rear body and propelling the caterpillar forward.

From the mechanical point of view, several modelling strategies for crawling systems, such as those inspired by caterpillar locomotion, often refer to continuous and discrete mechanical responses [41–45]. In this context, in [38], we proposed a discrete, lumped mass, one-dimensional (1D) mechanical model deliberately essential to capture the fundamental aspects of adhesion and cumulated elastic energy of caterpillar locomotion. In detail, the locomotion trigger has been modelled as an inelastic contraction localized at the terminal subunit. This captures how the terminal segment acts as a pivot point, generating an anterograde wave that propagates through the invertebrate's soft body and propels it forward [27,46,47]. The interaction with the substrate is modelled using horizontal Winkler-like constraints [48–50], whose elasticity competes with that of the animal's body.

While in our previous research [38,51], we examined and measured the adhesion forces of caterpillars on substrates, such investigations have been largely limited to wooden surfaces, primarily to trace back their behaviour within the natural environments typically explored by the animal. Yet, a crucial aspect for bioinspired design lies in extending this understanding to a wider variety of substrates. Indeed, for the development of advanced caterpillar-inspired robotic systems, it is essential to assess locomotion not only under natural conditions but also across ‘artificial’ surfaces that present distinct physical properties and roughness patterns with respect to the wood. In order to address this gap, the present work explores caterpillar locomotion on different substrates, thereby providing insights that may be crucial for glimpsing improved robotic prototypes. To do this, we recovered our 1D mechanical model in order to evaluate its capability to reproduce caterpillar gait across different substrates, experimentally observed through a series of ad hoc conceived laboratory tests. Notably, by equipping caterpillars with additional stiffening armour and additional masses, we showed that locomotion can be made possible on any substrate, also on previously non-walkable supports. Building on these findings, we showed that our previously developed model [38,51] can be used not only to accurately capture the experimental average velocities of the soft invertebrate, but also as a versatile mathematical tool to guide the design of caterpillar-inspired robots. In detail, by properly tuning its parameters, this theoretical framework also validated through experimental tests enables the development of systems capable of overcoming obstacles and moving on substrates where natural caterpillars would fail.

The paper is structured as follows: §2 briefly reviews linear elastostatic and dynamic modelling strategies from a design perspective, before providing a comprehensive discussion of experimental observations of caterpillars on different substrates. Section 3 presents a proof-of-concept that experimentally validates the idea of enhancing locomotion by altering stiffness/adhesion ratios equipping caterpillars with artificial armour and applied weights that increased the effective friction, in this way allowing the animal to walk on material substrates where otherwise the locomotion would be impeded. Section 4 provides the strategy for designing engineered bioinspired devices that mimic caterpillar locomotion, overcoming the limitations of the ‘natural’ soft animals. Finally, §5, conclusions and future perspectives, summarizes the key aspects and main findings, also envisaging potential developments for the ongoing research.

This study advances the understanding of caterpillar locomotion and provides key design criteria for soft robots navigating diverse terrains. We definitively demonstrate how the mechanical principles of caterpillar locomotion can be mimicked and transferred to a wide range of supports and prototypes.

## 2. Modelling and evidence of substrate-dependency caterpillar locomotion

In order to define a design strategy aimed at optimizing the locomotion performance of bioinspired prototypes, after having recalled the mechanical framework that captures the essential issue of the caterpillar movement, we examine its adaptability and behaviour on different substrates. Therefore, in the next subsection, we revisit the linear equations previously introduced in our research [38,51], with the goal of highlighting their utility in describing the fundamental principles of the soft-bodied locomotion and also showing their applicability for design purposes. In the second subsection, we extend the investigation to consider the actual behaviour of the caterpillar across different substrates, both experimentally and theoretically. This dual approach enables, on one hand, the identification and emulation of the advantageous aspects of caterpillar locomotion, and on the other hand, the possibility to overcome specific limitations encountered on particular substrates by evaluating how design parameters can also be modulated in order to gain locomotion on substrates where ‘natural’ caterpillars cannot move.

### 2.1. Fundamentals for modelling caterpillar locomotion

To trace back the key mechanical features of caterpillar locomotion, we first recover the 1D discrete lumped mass system provided in [38,51] and reported in figure 1. In detail, body subunits are represented by discrete mass elements linked in series via elastic springs that emulate the body's elasticity. Adhesion to the substrate is modelled using Winkler-type springs, reproducing the abdominal prolegs, which play a dominant role throughout the crawling cycle compared to the thoracic legs, whose contribution is considered negligible. To simulate the attachment and detachment phases of the prolegs, we implemented a linear elastic constitutive model that includes a threshold displacement $u_{y}$ during the tensile phase, corresponding to a specific level of adhesion on the substrate.

**Figure 1.**
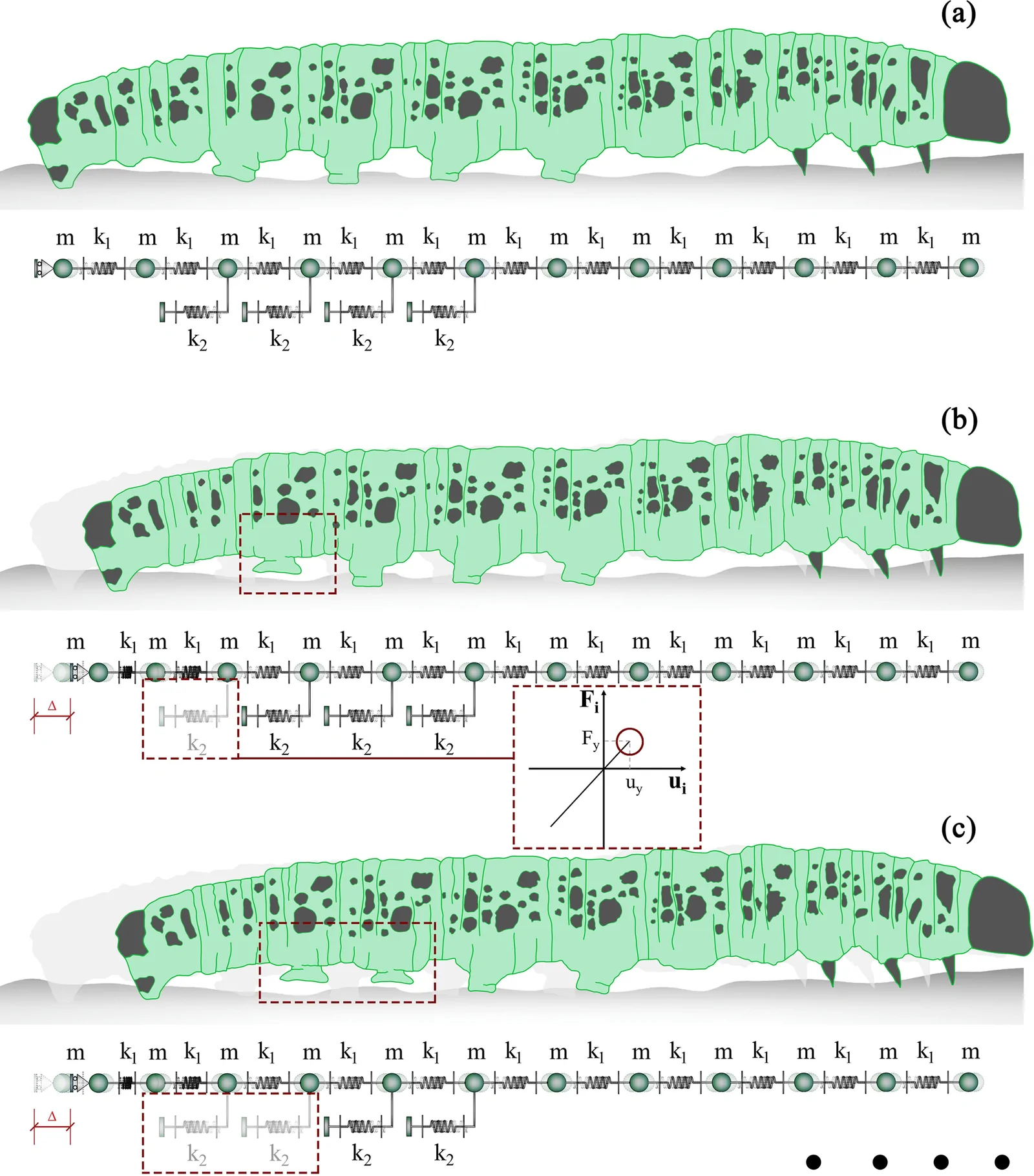
Sketch representing the progressive steps of the caterpillar locomotion and its relative mechanical model: (a) lumped mass system reproducing the several body segments at a stance phase; (b) muscle contraction Δ activates locomotion and induces the detachment of the first proleg, modelled by means of an adhesion threshold-based constitutive law; (c) schematic of the second substep, corresponding to the detachment of the second abdominal proleg. The crawl cycle continues until all four abdominal prolegs detach and reattach after the whole caterpillar body moves by a distance Δ.

The 1D mechanical system depicted in figure 1a, activated by an inelastic displacement applied at the terminal proleg, here denoted Δ, can be analysed using standard methodologies within the context of solid mechanics. As illustrated in figure 1a, the Lagrangian coordinates, $u_{i}(t)$, represent the horizontal displacements of each lumped mass $m_{i}$, with the displacement of the terminal mass, $u_{1}(t)$, prescribed to be equal to Δ. The total elastic potential energy $U_{\text{L}}$ of the lumped mass discrete structure shown in figure 1 can be expressed in the linear framework as follows:

(2.1) $$\begin{array}{rl} U_{\text{L}}= & \frac{1}{2}\sum_{i=2}^{n-1}k_{2}[i]\left(u_{i}(t)\right){}^{2}+\frac{1}{2}k_{1}\sum_{i=2}^{n-1}\left(u_{i+1}(t)-u_{i}(t)\right){}^{2}+\\ & +\frac{1}{2}k_{1}\left(u_{2}(t)-\Delta \right){}^{2},\;\;n=12,\\ \text{where}\;\;k_{2}[i]= & \left\{ \begin{array}{cl} & k_{2,}\;\;\;\text{if}\;\;\;i=\{3,4,5,6\},\;\;\\ & 0,\;\;\;\text{otherwise}, \end{array}\right.\text{where}\;\;u_{i}\leq u_{y}, \end{array}$$

where $k_{1}$ and $k_{2}$, respectively, are the stiffness of the inter-masses and Winkler-like springs. Note that the time dependence of the displacements $u_{i}(t)$ makes sense exclusively under dynamic conditions, which are subsequently discussed. In addition, the contribution of the thoracic legs in shaping the 1D caterpillar locomotion has been considered negligible [38,51].

By minimizing $U_{\text{L}}$ with respect to the unknown displacements (ranging from $u_{2}(t)$ to $u_{12}(t)$) and by introducing the stiffness ratio α as $k_{2}\;{k_{1}}^{-1}$, the equation corresponding to the ith degree of freedom (d.f.) can be obtained as follows:

(2.2) $$k_{1}(u_{i}-u_{i-1})+\alpha \;k_{1}[i](u_{i})-k_{1}(u_{i+1}-u_{i})=0.$$

The entire crawling motion is modelled as a sequential detachment of prolegs, governed by an adhesion threshold applied to Winkler-like springs. The process starts with all abdominal prolegs attached to the substrate. When an inelastic displacement Δ is applied, it generates a stress distribution across the structure, causing at least one spring to exceed the adhesion threshold $u_{y}$ and detach. The mechanical system's configuration is then updated by removing the spring that has surpassed $u_{y}$, and internal forces are recomputed using the same displacement Δ. This iterative procedure continues until all the prolegs have detached. Once the crawling phase is complete, all Winkler-like springs adhere again to the substrate to simulate the active reattachment of the prolegs. This stepwise update of the mechanical configuration automatically inoculates nonlinearity into the model and requires the use of the custom parametric algorithm described in [38], which allows updating the mechanical configuration at any step.

In order to trigger and maintain stable the step-by-step locomotion mode, the inelastic displacement Δ must be properly calibrated to avoid interpenetration between adjacent lumped masses, thereby ensuring geometric compatibility, as detailed in [38]. To achieve this, following some algebra reported in [38], the inelastic displacement required to enable the progressive detachment of all abdominal prolegs and to complete the whole crawl cycle—here denoted $\tilde{\Delta }$—can be numerically determined as a function of the adhesion threshold $u_{y}$ and the stiffness ratio α, as follows:

(2.3) $$\begin{array}{rl} & \tilde{\Delta }≃u_{y}\left\{ \begin{array}{clclc} & \frac{3\alpha^{3}+13\alpha^{2}+12\alpha +1}{\alpha^{2}+3\alpha +1}, & \; & \text{if }0<\alpha \leq 0.130, & \\ & \frac{4\alpha^{2}+9\alpha +1}{\alpha +1}, & \; & \text{if}\;\;0.130<\alpha \leq 3 & \\ & (5\alpha +1), & \; & \text{if }\alpha >3, & \end{array}\right.,\\ & \text{with}\;\tilde{\Delta }\leq l_{0}, \end{array}$$

where $l_{0}$ is the resting length of the inter-mass springs. The piecewise function $\tilde{\Delta }$ equation (2.3) defines additional constraint conditions that limit the range of possible inelastic displacement values, depending on the stiffness ratio of the system.

The piecewise expression equation (2.3) serves not only as a geometric compatibility condition but also as a valuable design tool, enabling the tuning of the stiffness ratio α and the adhesion threshold $u_{y}$ to control the inelastic displacement $\tilde{\Delta }$ required to trigger the motion of the system.

In order to validate the elastostatic assumptions made, we extended the model to the system's dynamic response by explicitly incorporating inertial contributions and dissipative terms, following standard analytical dynamics approaches [52–54]. Viscous effects have been modelled through linear dashpots arranged in parallel with the inter-mass springs, resulting in a sequence of Kelvin–Voigt (KV) units connected in series, as depicted in figure 2.

**Figure 2.**
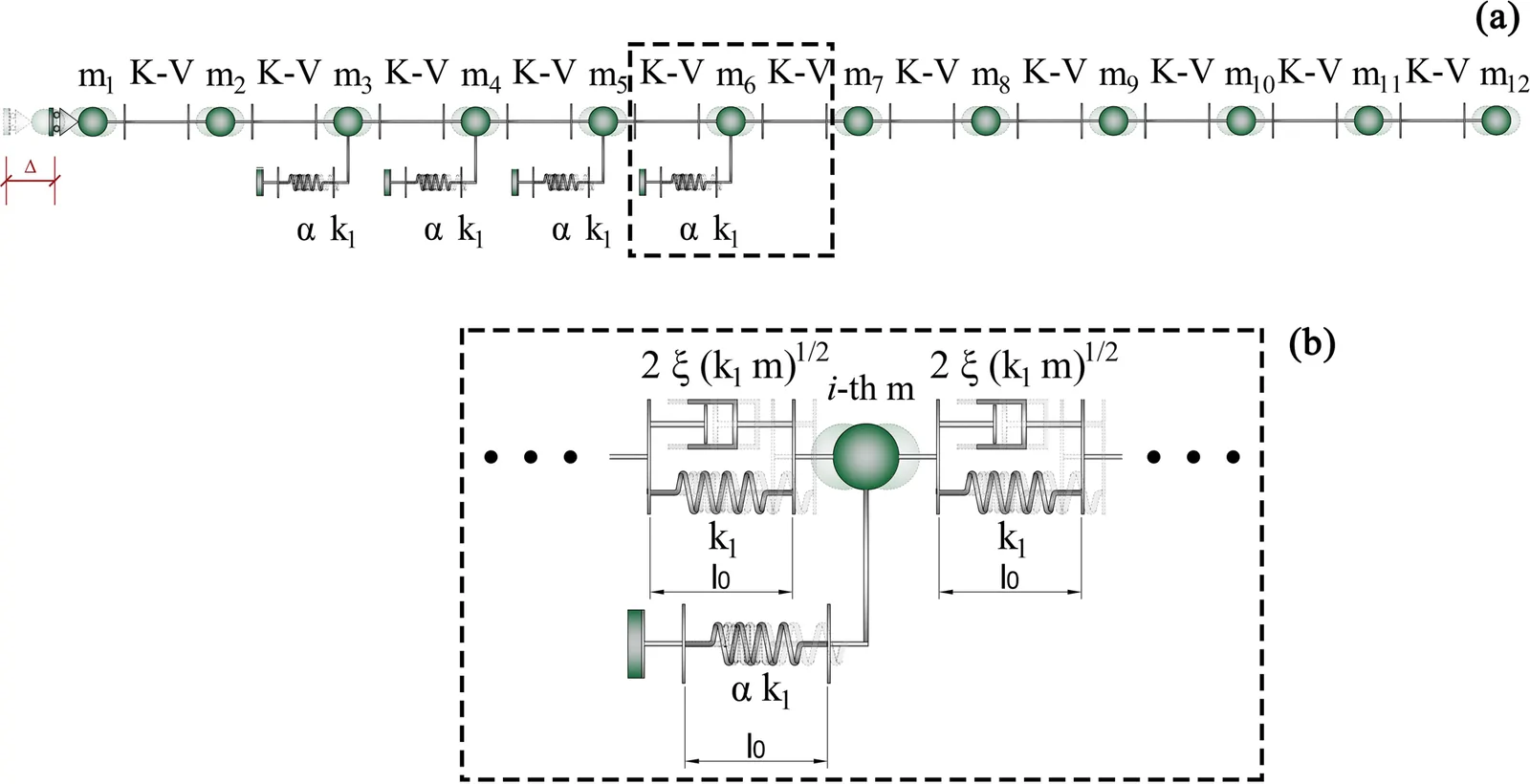
Schematic representation of the discrete dynamic lumped-mass model used to capture inertial and dissipative effects in caterpillar locomotion. Linear viscous dashpots are arranged in parallel to the inter-mass springs, which represent the body elasticity.

As reported in [38], the governing equation for the general ith lumped mass can be written as

(2.4) $$\begin{array}{rl} & k_{1}(u_{i}(t)-u_{i-1}(t))+\alpha k_{1}[i]u_{i}(t)\\ & -k_{1}(u_{i+1}(t)-u_{i}(t))+m{\ddot{u}}_{i}(t)\\ & =-2\xi \sqrt{k_{1}m}({\dot{u}}_{i}(t)-{\dot{u}}_{i-1}(t))\\ & +2\xi \sqrt{k_{1}m}({\dot{u}}_{i+1}(t)-{\dot{u}}_{i}(t)), \end{array}$$

where the overdot denotes differentiation with respect to time and ξ represents the non-dimensional damping ratio, defined as the damping coefficient of the KV element normalized by the critical damping value. Experimental observations on caterpillars indicate an overdamped behaviour, with ξ typically ranging between 1 and 5 [44].

For the system composed of 11 coupled differential equations in the unknown displacements $u_{i}(t)$ (from $u_{2}(t)$ to $u_{12}(t)$), homogeneous boundary conditions have been imposed. Consistently, with the formulation of the elastostatic model, the displacement $u_{1}(t)$ has been prescribed to coincide with the trigger $\tilde{\Delta }$.

Importantly, the stiffness terms $k_{1}$, $k_{2}$ and α, the resting length $l_{0}$, the mass m and the damping ratio ξ are intrinsic, substrate-independent parameters dictated by the caterpillar biomechanics. By contrast, the displacement threshold $u_{y}$ depends directly on the surface morphology; thus, Δ is evaluated as a function of both $u_{y}$ and α (equation (2.3)). Consequently, once $u_{y}$ is calibrated for a specific surface and the intrinsic mechanical substrate-independent parameters are *a priori* provided, the model may serve as a predictive tool capable of capturing the experimental displacements and velocities of the caterpillar.

In [38], we showed that, with the sole exception of the transient phase, the contribution of the inertial and dissipative effects can be considered negligible to reproduce the fundamental aspects of locomotion in the steady-state stage.

### 2.2. Influence of substrates: evidence from laboratory

Understanding how locomotion strategies adapt to varying environmental conditions is essential for both biological insights and bioinspired design. In this context, we investigated the response and the sensitivity of caterpillars to different substrates, aiming at identifying the key features that affect their performance and to derive principles applicable to the development of soft robotic systems.

As mentioned above and demonstrated in [38], adhesion plays a key role in caterpillar locomotion. To further explore how substrate properties—specifically surface rigidity and roughness—affect adhesive performance and locomotor behaviour, we conducted experiments on several distinct surfaces: birch wood, steel, slate and glass. Specifically, we compared locomotion on a wooden surface—representative of the typical environment where caterpillars move—with other substrates, in order to assess their adhesion and propulsion on unfamiliar supports.

Despite the apparent biological irrelevance of analysing caterpillars on substrates different from their natural ones, such investigations prove valuable from different points of view. They may not only enhance our understanding of the eventual geometric compatibility between the prolegs microstructures and the wooden patterns, but may also open new avenues for the design and development of bioinspired devices. Both quantitative and qualitative aspects of locomotion were in-depth analysed, by also measuring characteristic times and velocities. To do this, in the first instance, the experimental set-up has been ad hoc conceived to enable direct comparison across the steel and the wooden surfaces, the latter having already been tested in our previous works [38,51].

As already described, in figure 3, a series of time-step snapshots documents the movement of the caterpillar on two different supports: steel and wood. Initially, the caterpillar is positioned entirely on the steel surface (at time step 0 s). It then moves autonomously—without any external *stimulus*—towards and on to the wooden substrate. During the initial phase (0–10 s), the caterpillar progresses steadily over the steel, suggesting that it can somehow adapt its adhesion and effective muscle contraction starting from the pivot point. Importantly, as depicted in figure 6, the average velocity of the caterpillar moving on the steel support is much lower than the one registered on the wooden substrate. Between approximately 12 to 17 s (see figures 3 and 6b), as the anterior segments begin to interact with the wood, a temporary deceleration is noticeable owing to the increased surface roughness and altered contact mechanics. This transition phase may require the caterpillar to adjust its gait and recalibrate its inelastic muscle contraction to induce forward motion. Once fully engaged with the wooden surface (from approx. 20 s onward), the caterpillar resumes more uniform locomotion, although with slightly altered body proleg placement patterns compared to its motion on steel, achieving higher velocity values, as synoptically reported in the caption of figure 6b. These laboratory observations suggest that both the adhesion grip and the pivot action exerted by the terminal proleg influence the locomotion modes in terms of inelastic muscle contraction, frequency of the crawl-cycle and average velocity of the system. Therefore, as a possible challenge for the design of bioinspired systems, by properly tuning the mechanical and geometrical parameters into the model recalled in §2.1, the limitations in terms of velocity and gait of the natural caterpillar locomotion can be overcome and can be incorporated into the caterpillar-like robotic device, thus including the possibility to move on other substrates, by still preserving the main pecularities of the motion, such as stability and periodicity.

**Figure 3.**
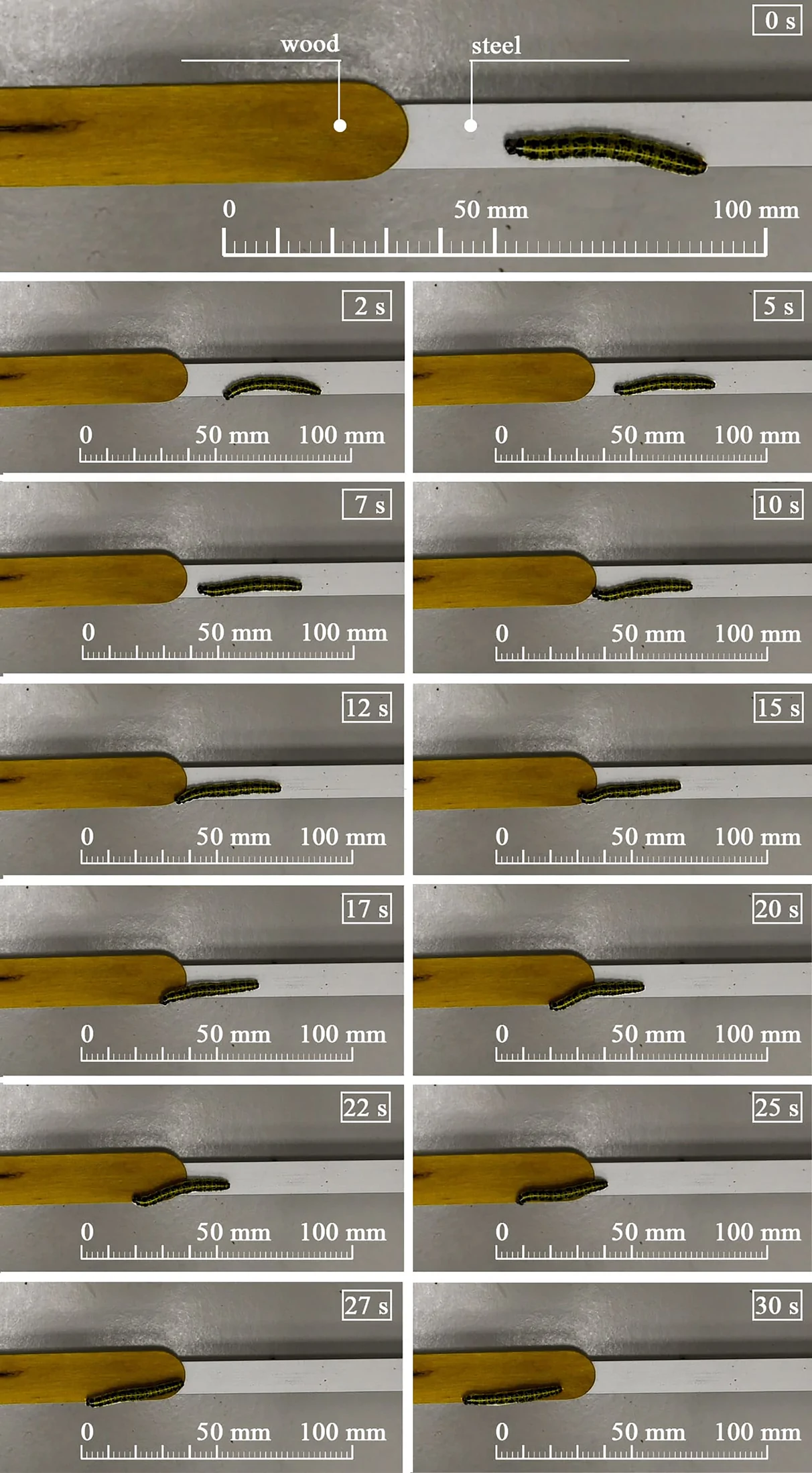
Time-step snapshots capturing the locomotion of *Pieris brassicae* on two different substrates: steel and birch wood. No external stimulus was applied, allowing the caterpillar to move spontaneously in a 1D direction from the steel surface to the wooden substrate.

**Figure 4.**
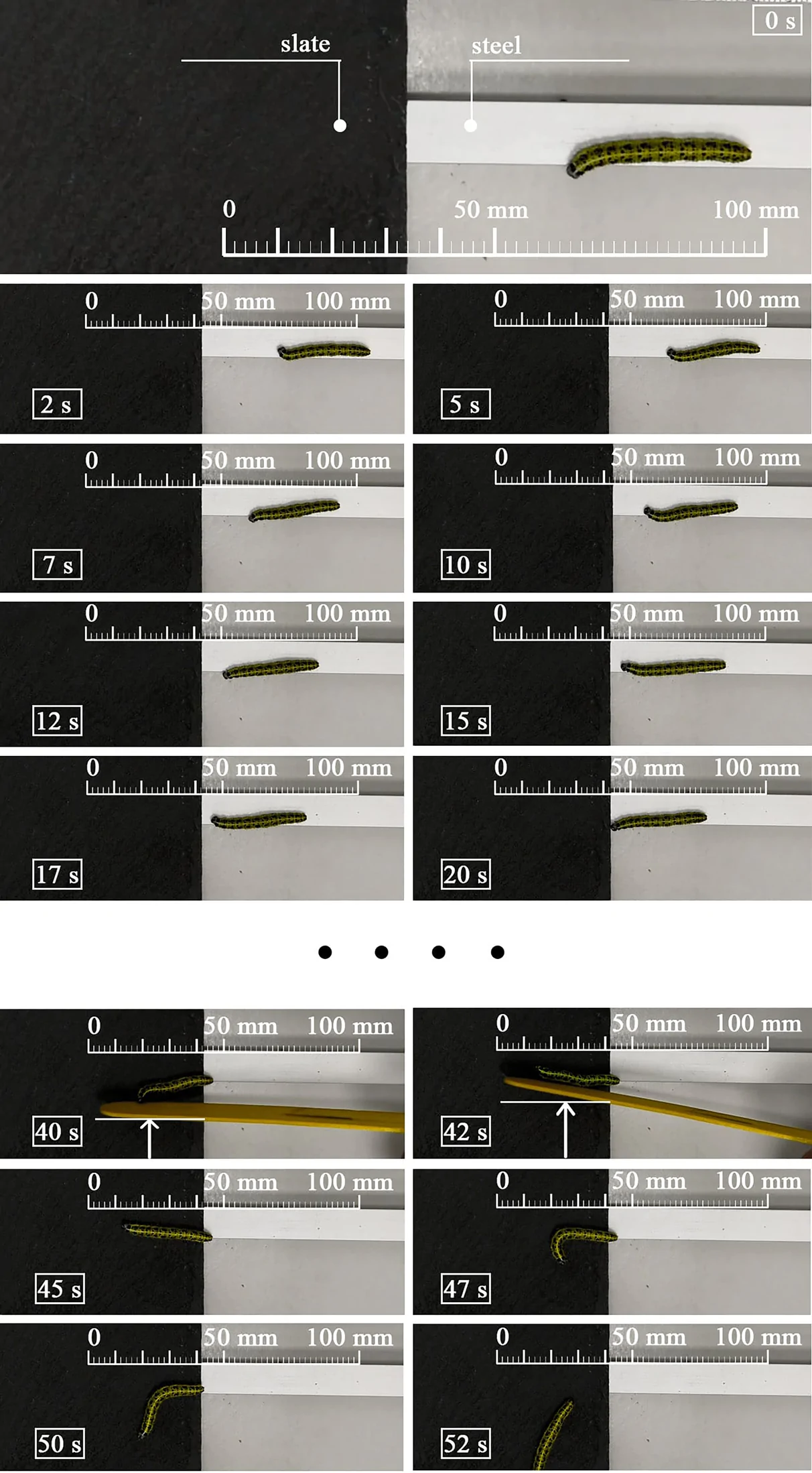
Time-lapse sequence showing the locomotion of *Pieris brassicae* on a two-material substrate composed of steel (right) and slate (left). The caterpillar begins on the steel side and spontaneously moves towards the slate without external *stimulus*. At 40--42 s, a wooden element (highlighted by white arrows) is used to reposition the caterpillar in a horizontal alignment with respect to the slate surface pattern. Nevertheless, the caterpillar reorients and resumes its original direction of motion, following the substrate pattern, as one can observe in snapshots at time 50 and 52 s.

**Figure 5.**
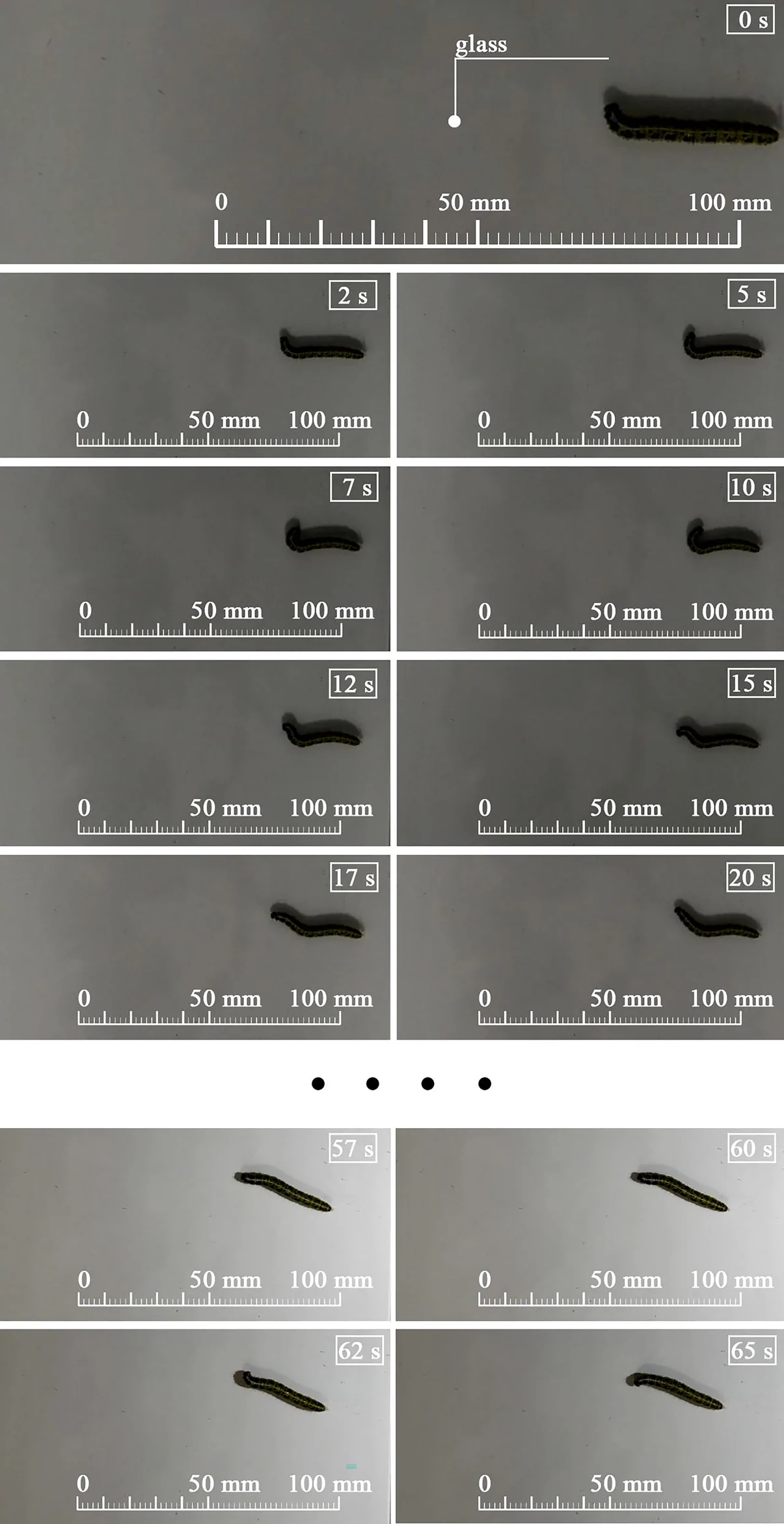
Time-lapse sequence of *Pieris brassicae* crawling on a glass substrate. The caterpillar moves spontaneously from right to left without external stimuli, yet after about 65 s, it exhibits only minimal net displacement, suggesting the motion on this substrate may be particularly hard for the animal.

**Figure 6.**
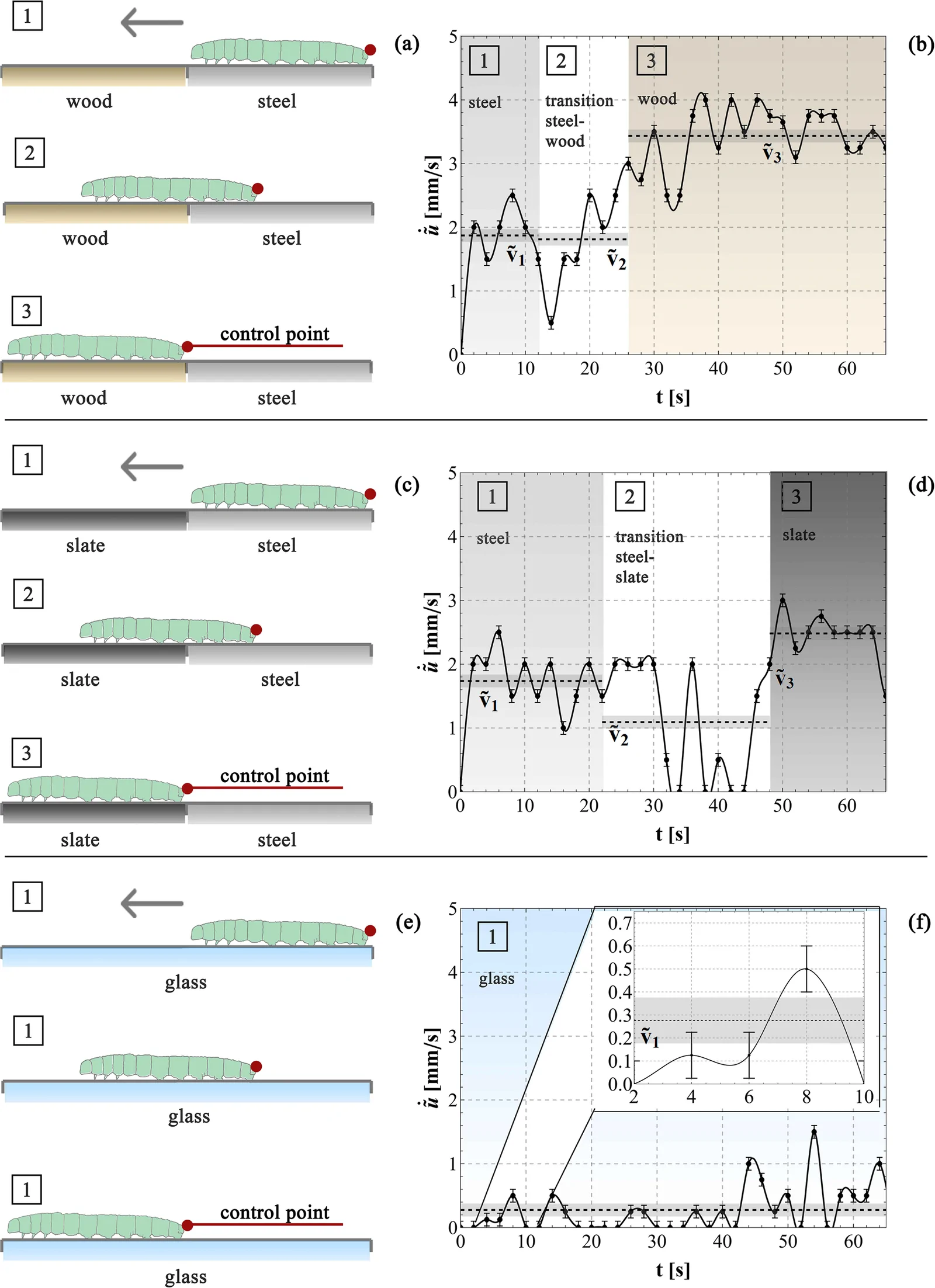
(a) Sketches showing the caterpillar moving on different substrates correlated to the corresponding registered average velocities on the right-hand column. The red dot marks the control point used for tracing back the average displacement of the soft invertebrate during locomotion, analysing the subsequent frames of the movie. No external *stimulus* was applied for all the laboratory tests; movement occurred spontaneously. By numerically differentiating the average displacement measures over time, the average velocity histories $\dot{u}$ can be obtained and their plots, referred to as steel-wood, steel-slate and glass surfaces, are reported, respectively, in panels (b), (d) and (f). Data are plotted over a time length of 65 s, beyond which the locomotion trends are almost stabilized and repetitive. By reiterating the experimental tests for four trials, a variance of about 10% of the values is registered and here graphically indicated through the error bars reported in all the plots. With reference to the subfigure (b), three regions are highlighted: (1) locomotion on steel, (2) transition phase (partially on both substrates) and (3) locomotion on wood. The average velocities (±10% underlined by the grey transparent region) in each range are ${\bar{v}}_{1}=1.87$ mm s^–1^, ${\bar{v}}_{2}=1.81$ mm s^–1^ and ${\bar{v}}_{3}=3.29$ mm s^–1^. In addition, with respect to the subplot (d), the average velocities, respectively, for each zone are ${\bar{v}}_{1}=1.74\pm 10\%$ mm s^–1^, ${\bar{v}}_{2}=1.18\pm 10\%$ mm s^–1^ and ${\bar{v}}_{3}=2.48\pm 10\%$ mm s^–1^. Finally, with respect to the glass surface, the average velocity is very low, reaching the value of ${\bar{v}}_{1}=0.28\pm 10\%$ mm s^–1^.

Within this perspective, we investigate the motion of the animal on further supports. In detail, figure 4 shows a time-lapse sequence of the spontaneous locomotion of a *Pieris brassicae* caterpillar (total resting length 38 mm) across two consecutive substrates, consisting of slate and steel. Initially, the caterpillar is entirely positioned on the steel surface. Over time, it autonomously progresses towards the slate support, exhibiting a clear directional preference, guided by the surface pattern. In fact, at time 40–42 s, an external wooden element is used to reposition the caterpillar so that its body is aligned perpendicularly to the slate texture, with the aim of placing it in a horizontal orientation with respect to the prevalent inclination of the slate surface's pattern (at time 47--48 s). Despite this intervention, the caterpillar consistently and naturally reorients itself and resumes movement in the oblique direction, somehow indicating a robust and substrate-driven locomotion strategy. This persistent, mechanically driven movement, often observed in several biological and living systems [20,55,56], confirms the need of adopting a 1D modelling framework in which Winkler-type springs are introduced to modulate the interaction with the substrate, effectively constraining the locomotion to a preferred ‘track-like’ direction.

In addition, as reported in figure 5, further laboratory tests have been carried out on a glass substrate. Specifically, the time-lapse sequence documents the locomotion of *Pieris brassicae* larvae on a glass substrate. Each frame corresponds to a specific time point, beginning at 0 s and extending to 65 s, with spatial measurements provided by a millimetre scale displayed in every image. The caterpillar initially progresses from right to left, continuously exhibiting periodic cycles of body contraction and extension characteristic of the crawling behaviour. The sequence reveals a remarkably low net displacement over the full observation interval, compared to the other previous locomotion modalities. This limited motion is due to the lack of full adhesion of the soft-invertebrate prolegs on the glass surface, thus implying a significant reduction of the overall progression, which is estimated in the following as a reduction, respectively, of 91%, 89% and 85% with reference to wood, slate and steel.

Starting from the experimental tests carried out, the corresponding velocity profiles on the different substrates numerically derived from average displacement measurements are obtained and reported in figure 6. In particular, in each schematic of the locomotion (left-hand column), the caterpillar progresses spontaneously from right to left, while the red dot identifies the control point used for tracking its motion across consecutive video frames. The plots on the right-hand side quantify the average velocity $\dot{u}(t)$ as a function of time, obtained by the numerical differentiation of the displacement data recorder through the video. Interestingly, for both heterogeneous substrate combinations (steel–wood and steel–slate), the velocity histories reveal three distinct locomotion phases-zones: (i) movement on steel, (ii) a transition region in which the animal simultaneously interacts with both substrates, and (iii) movement that fully occurs on the second substrate. The corresponding velocity trends and average values indicate a clear substrate-dependent behaviour. Actually, movement on the smoothest surface—steel—consistently yields the lowest velocities. By contrast, if we disregard the oscillations observed in the transition area, locomotion on both wood and slate leads to higher average velocities, with wood showing a slightly greater increase, since it represents the animal's natural environment, where nature has probably optimized the geometric shape of the prolegs to allow efficient movement on that surface. However, laboratory observations on glass exhibit markedly different dynamics. As one can see in figure 6e,f, the velocity profile on glass is characterized by very low magnitudes throughout the entire test, with average velocities an order of magnitude lower than those recorded on the other substrates (e.g. 0.28 mm s^–1^ versus 3.29 mm s^–1^ on the wooden support). This finding confirms that movement on the glass surfaces is hard for the animal, probably owing to the lack of adhesion of the terminal and the abdominal prolegs.

Regarding the statistical robustness of the experimental results, it is important to note that the behavioural patterns of these soft invertebrates are highly consistent owing to their biological simplicity and standardized experimental conditions. While larger sample sizes are often adopted in ecological studies, our observations showed negligible inter-individual variability, suggesting that a high number of replicates are not strictly required to capture the fundamental locomotion trends. Nonetheless, to ensure statistical validity, we conducted at least four independent trials for each experimental condition. As evidenced by the minimal error bars presented in figures 6 and 8, the observed behavioural consistency confirms that these findings are representative and reproducible across the selected caterpillar species. A summary comparing the average velocities, displacement and velocity variances and the velocity reduction with respect to the wood substrate is listed in table 1.

**Figure 7.**
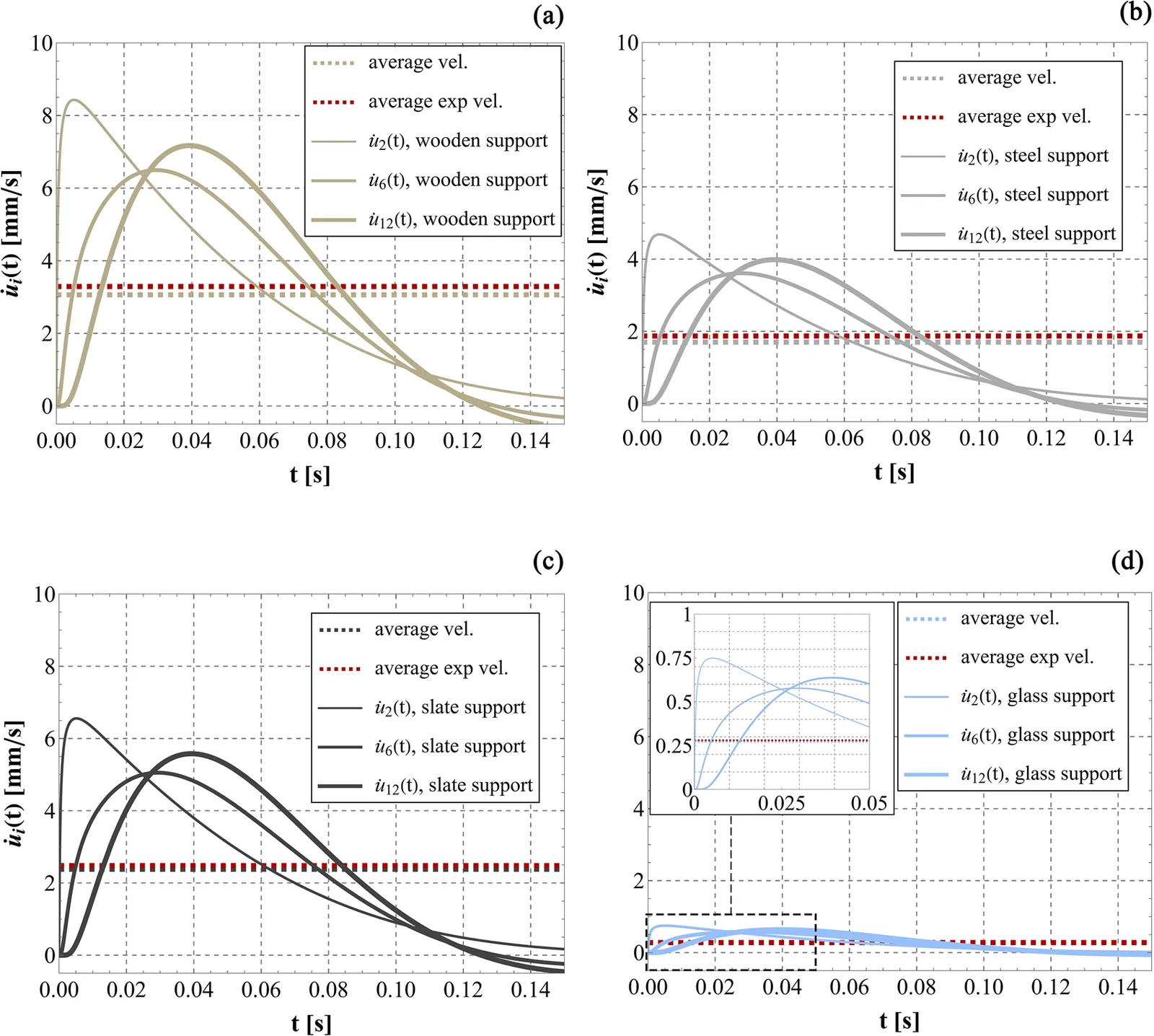
Numerical simulation results for selected d.f., obtained by calibrating the model through the tuning of the adhesion parameter $u_{y}$. For each support type (wood, steel, slate and glass), the simulated velocities ${\dot{u}}_{i}(t)$ are shown and compared with the reference average velocity. The curves illustrate the time evolution of the dynamic response, highlighting how the mechanical model may be useful for capturing the experimental observations reported in figure 5. For all the graphics, the parameters are set as: $k_{1}=5$ N m^–1^, α=0.01, $l_{0}=0.003$ m, $m=1\times 10^{-4}$ kg, ξ=5 and $\delta =4.89\times 10^{-4}$ m. The adhesion parameter $u_{y}$ has been fixed at: (a) $4.5\times 10^{-4}$ m (with numerical average velocity estimated at 3.06 mm s^–1^), (b) $2.5\times 10^{-4}$ m (with numerical average velocity estimated at 1.70 mm s^–1^), (c) $3.75\times 10^{-4}$ m (with numerical average velocity estimated at 2.40 mm s^–1^) and (d) $4\times 10^{-5}$ m (with average velocity estimated at 0.27 mm s^–1^). For all the plots, the red dashed lines indicate the average velocities experimentally registered in previous laboratory tests for the corresponding support.

**Figure 8.**
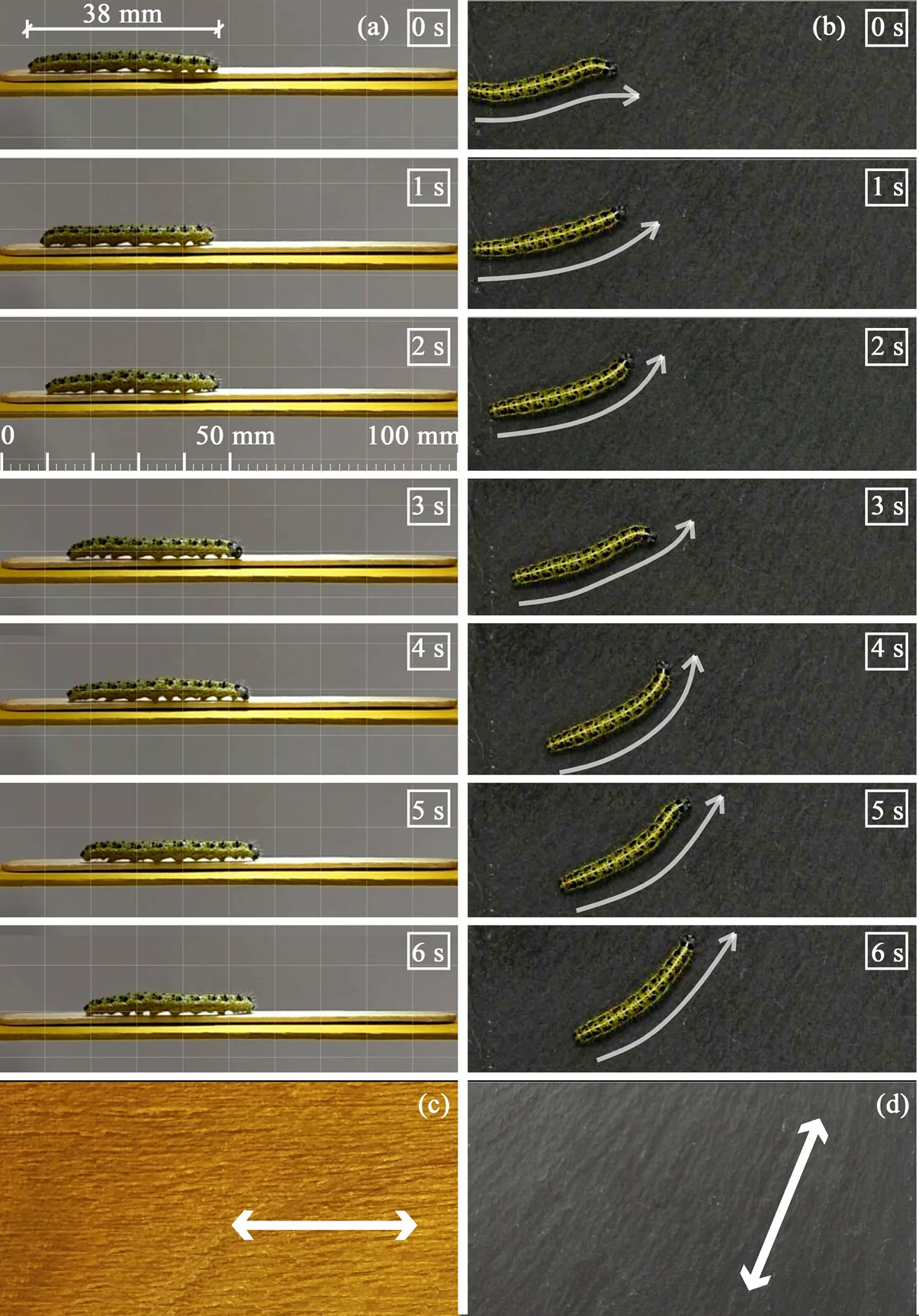
Time-lapse images showing the caterpillar locomotion of *Pieris brassicae* larvae (length 38 mm and weight 0.35 g) on two substrates with a focus on the substrate patterns: (a) wood and (b) slate. Across both columns, the behaviour of the caterpillar highlights the influence of substrate properties: (a) on wooden support, the crawler achieves consistent movement aligned with the longitudinal grain, as also detailed in the enlargment at panel (c); (b) on slate, it adapts its gait to follow the surface roughness, as highlighted by the grey arrow and in the in-zoom view of panel (d).

**Table 1.** Synoptic summary of the experimental observations related to caterpillar locomotion on different substrates reported in figure 6. While the average velocity has been evaluated as the time-integral average of the velocity history of a single recording test, the variance has been estimated from at least four independent trials.

substrate	average velocity $\mu_{\bar{v}}$ [mm s^–1^]	reduction with respect to wood	variance $\bar{\sigma }$ [-]
birch wood	3.29	–	≃10%
steel	1.81	45%	≃10%
slate	2.48	25%	≃10%
glass	0.28	91%	≃10%

To assess how effectively the mechanical model recalled in §2.1 reproduces the laboratory observations shown in figure 6, we report some numerical solutions for selected caterpillar subunits in figure 7. These results were obtained by providing as input the estimated mechanical parameters for the model and calibrating the adhesion parameter $u_{y}$ through a best-fit procedure to match the average experimental velocities recorded for each substrate (steel, wood, slate and glass).

In more detail, the average velocity ${\tilde{v}}_{i}$ has been estimated for each d.f. as the time-integral average of the velocity history over a single crawl substep, explicitly defined as

(2.5) $${\tilde{v}}_{i}=\frac{1}{\tau }\int_{0}^{\tau }{\dot{u}}_{i}(t)\;dt,$$

where τ is the characteristic time duration of a single crawl-substep, experimentally measured and approximately equal to 0.15 s. The final velocity value for each substrate was then computed as the arithmetic mean of the integral velocities across the d.f.

The plots report the time evolution of the simulated velocities ${\dot{u}}_{i}(t)$, which are compared with the reference average velocity. Quite trivially, from the employed model (see its fundamentals in §2.1), the amplitude of the dynamic response is scaled as a function of the threshold adhesion parameter $u_{y}$, whereas its frequency content—which depends on the mechanical parameters of the system (i.e. the caterpillar's mass and stiffness)—remains unchanged.

The results prove the substantial influence of the adhesion parameter $u_{y}$ on the dynamic response of the system. The best-fitting values enable the model to reproduce the characteristic response associated with each substrate, thereby confirming the capability of the proposed mechanical model to capture the trends observed in the laboratory tests.

As an ancillary result, we further investigated whether caterpillars reorient their locomotion based on substrate directionality; these findings are summarized in figure 8: the figure presents time-lapse sequences illustrating the crawling gait of *Pieris brassicae* larvae (length: 38 mm; weight: 0.35 g) on three distinct substrates: (i) wood, (ii) slate and (iii) glass. A combined qualitative and quantitative analysis is conducted to characterize the locomotor performance in relation to substrate properties, with particular attention to the preferential movement patterns and the subunit deformations. In more detail, in panel (a) of figure 8, locomotion on wood reveals a consistent and directional progression aligned with the grain of the substrate, indicating a preferential pattern driven by the anisotropy of the wooden support, as highlighted in panel (c) of figure 8. On this support, significant deformations are observed in the posterior terminal segment during the propulsion phase with stretches ranging from approximately 0.7 to 1.05, as documented by Argenziano *et al.* [57]. On slate (panel (b) of figure 8), the larva displays adaptive behaviour, actively adjusting its body posture to navigate following the oblique direction highlighted in the figure. In this case, the movement appears to be modulated by discontinuities in the surface topology, as indicated by the grey arrow and further detailed in the zoomed view in panel (d) of figure 8. This behaviour seem to suggest that caterpillars intentionally select to follow the preferential patterns of the substrate. In particular, in contrast to larger animals that exploit lateral movements and frictional anisotropy [22,58], as for instance snakes, caterpillar crawling is primarily constrained to the sagittal plane, driven by the mentioned cooperative interplay between body elasticity and proleg-substrate adhesion/friction. The substrate affects this locomotion in two fundamental ways. First, surface roughness dictates the effective friction and adhesion—depending on its match with the proleg setae—which can either facilitate periodic movement or arrest it entirely. Second, surface patterns and micro-morphological anisotropy can steer the larva's trajectory. When encountering curved well-defined patterns, as for the case of slate represented in figure 8, caterpillars adjust their path so that their sagittal plane of motion remains tangent to the physical pattern. Understanding these dual substrate effects may be particularly relevant for optimizing the design of bioinspired robots operating in geometrically complex and heterogeneous environments.

Starting from these results, in order to correlate the adhesion of caterpillars on the specific support, as a first step, we refer to the appropriate metrics conventionally adopted for quantitatively characterizing the substrate properties [59–61], that is, the characteristic roughness arithmetic average parameter and the root mean square roughness, here named, respectively, as $R_{a}$ and $R_{q}$ and whose expressions are recalled in what follows:

(2.6) $$\begin{array}{rl} & R_{a}=\frac{1}{L}\int_{0}^{L}|z(x)|\;dx\\ \text{and } & R_{q}=\sqrt{\frac{1}{L}\int_{0}^{L}z^{2}(x)\;dx,} \end{array}$$

where z(x) is the profile height along the horizontal x-axis and the sample length L. For the selected substrates, the numerical values of $R_{a}$ and $R_{q}$ have been extracted from the inherent literature and summarized in table 2 to provide a quantitative comparison of the surface topography. It is well-established that roughness parameters are not intrinsic material properties, but are instead predominantly determined by the manufacturing process, surface morphology and grain. Consequently, the values listed in the table 2 represent the typical ranges to account for a wide spectrum of production conditions. In addition, to correlate more specifically the caterpillar adhesion on the selected substrates, we performed a further surface morphology analysis using the scanning electron microscope (SEM) available in our laboratory. Representative micrographs of the investigated substrates together with the ones related to the abdominal proleg of the species *Pieris brassicae* are reported in figure 9.

**Figure 9.**
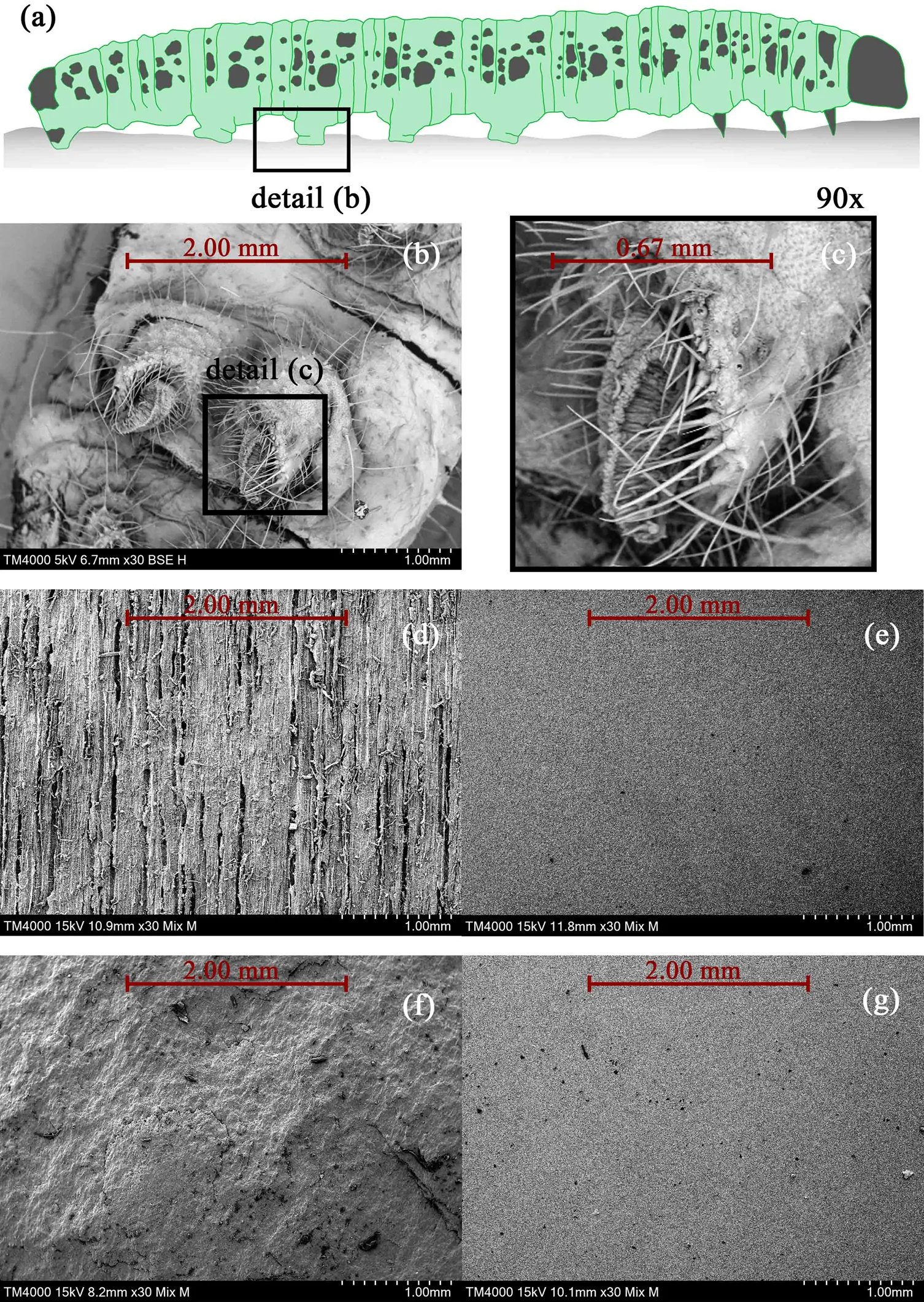
SEM analysis of larval features and surface substrates. (a) Schematic representation of the caterpillar with highlights for details (b) and (c) (the SEM snapshot (b) of the caterpillar is extracted and re-edited from our previous research paper [38]). In more detail, the subplot (b) reports the SEM micrograph of the abdominal prolegs with characteristic length of the distance between the two sucker-like portions approximately equal to 2 mm; the panel (c) refers to a 90× enlargement focusing on the *setae* of the abdominal proleg, being the average characteristic length of the single hair ∝250μm, while the distance between two ones is approximately ∝30μm. In panels (d–g), the SEM snapshots of various substrate surfaces are given: (d) wood, (e) steel, (f) slate and (g) glass.

**Table 2.** Typical surface roughness parameters for the substrates adopted for the experimental campaign deduced from the dedicated literature. Specifically, $R_{a}$ indicates the arithmetic average roughness, while $R_{q}$ refers to the root mean square roughness. Values are expressed in micrometers (μm).

substrate	$r_{a}$ (μm)	$r_{q}$ (μm)	literature references
birch wood (*Betula pendula*)	5.0–25.0	6.0–30.0	[59,62,63]
steel (polished to milled)	0.1–3.2	0.12–4.0	[64–66]
slate (natural cleft)	15.0–50.0	20.0–65.0	[67,68]
glass (float)	0.001–0.01	0.0015–0.015	[69–71]

As shown in figure 9d, the wooden substrate exhibits, at the scale relevant to the caterpillar prolegs, a pronounced periodic surface morphology characterized by a wavelength—defined as the distance between consecutive ridges and valleys—ranging from approximately 70 to 100 μm. This characteristic length scale is of the same order of magnitude as the measured average spacing between the larval *setae*, approximately estimated at 30 μm (figure 9b,c), thereby promoting an effective mechanical interlocking between the attachment structures and the substrate.

By contrast, a similarly well-defined periodic pattern is not observed for the slate substrate with respect to the wooden one. Nevertheless, macroscopic inspection at 30× magnification (figure 9f) reveals a surface roughness that, in agreement with the quantitative measurements listed in table 2, is considerably more similar to that of wood than to the steel and glass substrates, which appear notably much smoother, as one can immediately recognize from figure 9e,g. These observations appear consistent with the experimental locomotion results, which show that smoother surfaces provide less effective attachment, leading to reduced adhesion and consequently lower locomotion speeds. However, we note that, as extensively discussed in our previous work [38]—which presented a comprehensive experimental characterization of caterpillar adhesion—the adhesion effect, represented in the present model by the single parameter $u_{y}$, should be regarded as the macroscopic outcome of several concurrent physical mechanisms. These include microscopic van der Waals interactions, suction generated by the cup-shaped geometry of the prolegs and increased friction arising from the mechanical interlocking between the proleg *setae* and surface asperities. From this perspective, this latter mechanism, which is directly linked to the geometric compatibility between the prolegs and the substrate, is one component of the complex overall process that gives rise to adhesion. Consequently, the parameter $u_{y}$ captures the overall adhesive response resulting from the combined action of these mechanisms, rather than representing any individual contribution.

## 3. Empowering caterpillar locomotion: proof-of-concept

In order to investigate the possibility of artificially enhancing caterpillar locomotion, further experimental tests were performed.

The rationale behind these experiments is twofold: inducing a structural stiffening of the soft invertebrate and increasing substrate-animal adhesion by modifying caterpillar weight, thereby raising the resulting friction coefficient. To achieve these goals without altering the natural morphology of the prolegs, we adopted the simplest strategy, thus equipping the soft vertebrate with an artificial polymeric, i.e. polyvinyl chloride (PVC), armour stiffening—which increased its structural rigidity by 150--200%—and also weighting it with external lead masses, each one of about 0.30 g, thus reaching approximately three times the original caterpillar weight.

As an initial step, the locomotion performance in this modified configuration was evaluated on a wooden substrate, representing a natural walkable support for caterpillars. As the PVC stiffening armour is mounted on the sole anterior segment of the animal (figure 10), the crawler achieved an average longitudinal velocity ${\bar{v}}_{1}$ of 3.42 mm s^–1^, consistent with the one reached by caterpillars in natural conditions (figure 6b), thus confirming the model hypothesis that the anterior portion and the thoracic legs are not involved in locomotion. However, introducing an external payload—specifically a 0.37 g spherical lead mass placed only on the anterior segment—resulted in a performance reduction, decreasing the average velocity to 2.21 mm s^–1^, as shown in figure 10a,b. Therefore, when both the artificial stiffening and the additional weight were applied exclusively to the anterior subunits, no appreciable changes in the overall locomotion patterns and characteristic times/frequencies of the crawling cycles were observed, with the sole difference that by weighting the animal with an additional mass, a slight reduction of the velocity histories occurred.

**Figure 10.**
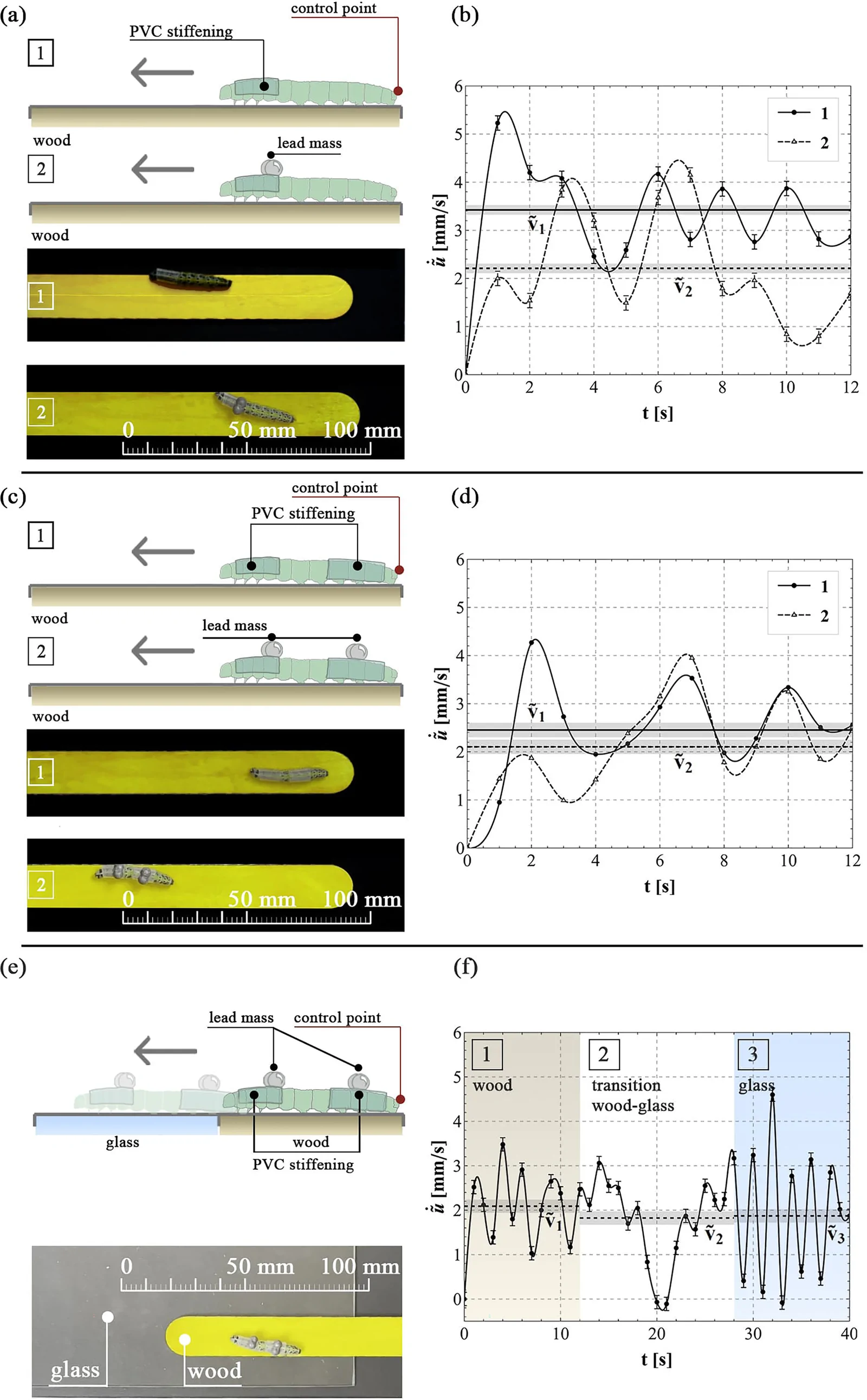
Experimental assesment of augmented caterpillar locomotion: (a) evidence of anterior segments not involved in locomotion (${\tilde{v}}_{1}=3.42$ mm s^–1^ and ${\tilde{v}}_{2}=2.21$ mm s^–1^), (b) increasing stiffness and adhesion on already optimal supports like wood slows down the velocity (${\tilde{v}}_{1}=2.45$ mm s^–1^ and ${\tilde{v}}_{2}=2.10$ mm s^–1^) and (c) increasing stiffness and adhesion on glass allowing locomotion (average velocities for the wood, transition and glass phases, respectively, equal to ${\bar{v}}_{1}=2.09$ mm s^–1^, ${\bar{v}}_{2}=1.85$ mm s^–1^ and ${\bar{v}}_{3}=1.87$ mm s^–1^). The experimental tests were repeated for four trials for each substrate, registering a maximum variance of about 15% in terms of average velocities, graphically highlighted through the error bars reported in all the plots. The corresponding variance of 15% is reported in the average velocities and indicated through a grey transparent area.

To further investigate the effect of structural rigidity, a double PVC armour configuration (both on the anterior and on the posterior body segments) was subsequently tested (figure 10c,d). The increased stiffness of this set-up led to a reduced average velocity of 2.45 mm s^–1^. Furthermore, by adding masses to both anterior and posterior segments with a total payload of 1.08 g, the average velocity still decreased to 2.10 mm s^–1^. As expected, on substrates like natural wood, where adhesion and animal elastic compliance are somehow optimal, the above-mentioned alterations of the natural setting hinder locomotion, demonstrating how the balance among structural elasticity, weight and adhesion determines the locomotion outcome.

Finally, the crawler was experimentally evaluated, forcing the artificially armoured and weighted caterpillar to move across different surfaces, i.e. from wood to glass (figure 10e,f). As the animal stayed on the wooden support at the early stage, the caterpillar proceeded with the average velocity of ${\bar{v}}_{1}=2.09$ mm s^–1^ already registered in the case 2 of figure 10e,f. Subsequently, as it crossed the boundary between the two substrates, the velocity dropped to ${\bar{v}}_{2}=1.85$ mm s^–1^ owing to uneven contact and grading surface conditions, this reduced velocity then stabilized at approximately ${\bar{v}}_{3}=1.87$ mm s^–1^ on the glass surface, which is surprisingly one order of magnitude higher than the one registered for caterpillars on glass without armour, as it emerges by comparing figure 6e,f and figure 10e,f. More specifically, regarding this last caterpillar configuration with the PVC stiffening armour and two mass beads, a 36.5% reduction in average velocity on wood is accompanied by an approximate 570% increase in performance on glass with respect to its unequipped counterpart, as summarized in table 3.

**Table 3.** Summary of the experimental observations from figure 10e,f. The locomotion performance of the caterpillar, artificially modified with a PVC stiffening armour and two mass beads, is analysed. Specifically, its average velocities are compared to those of the natural, unequipped caterpillar on the corresponding substrates. Also in this case, four independent trials have been carried out for each substrate.

substrate	average velocity $\mu_{\bar{v}}$ [mm s^–1^]	comparison with table 1	variance $\bar{\sigma }$ [-]
birch wood	2.09	−36.5%	≃15%
glass	1.87	+570%	≃15%

These findings highlight how insights gained from the theoretical locomotion model might be used for providing ad hoc modifications of body elasticity and adhesion in caterpillars to enhance their crawling performance and even allowing them to move on normally non-walkable substrates.

## 4. Envisioning strategies for optimizing locomotion on substrates

Based on these considerations and on the performed experimental tests discussed in the previous section, our goal is to devise strategies that overcome the limitations observed in ‘natural’ caterpillars, thus envisaging some potentially more efficient and controllable bioinspired devices. Through our theoretical model and experimental tests, we have identified the specific combinations of adhesion conditions and body elasticity required for effective locomotion across different substrates. By appropriately tuning these mechanical parameters, we may be able to design such engineered systems, ensuring effective propulsion even for substrates where natural caterpillars cannot move. To support this approach, we recover the geometric compatibility equation (2.3) graphically represented into the map of figure 11 as a tool to guide the design of artificial caterpillar-inspired robots. Therefore, by identifying the compatibility region—where locomotion is made possible and no interpenetration between adjacent segments occurs—we can *a priori* define the mechanical and geometric characteristics of the caterpillar-like prototype. In our framework, we associate the displacement threshold $u_{y}$ with the selection of the substrate. As emphasized in §2.2, while $u_{y}$ is primarily governed by the substrate geometry, it also encapsulates the interplay of multiple concurrent mechanisms. These include microscopic van der Waals interactions, suction generated by the cup-shaped prolegs and friction arising from mechanical interlocking between the caterpillar *setae* and surface microasperities. All these contributions are condensed into this single adhesion threshold. Consequently, the greater normal force and friction induced by the added bead masses in the artificial caterpillar configurations directly strengthen the mechanical interlocking—an effect that is macroscopically absorbed by $u_{y}$.

**Figure 11.**
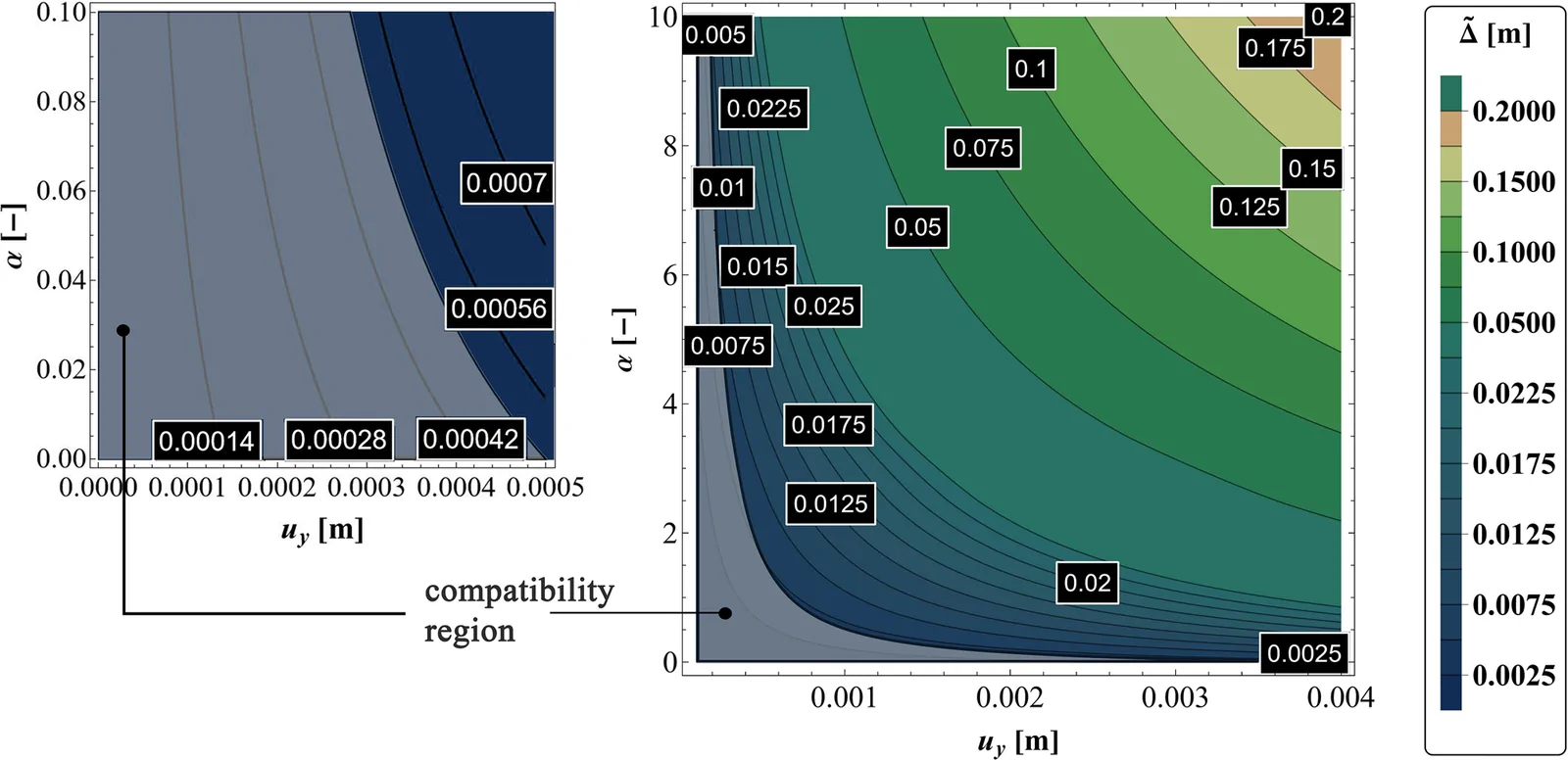
On the right-hand side, contour plot of the inelastic contraction $\tilde{\Delta }$ as a function of the threshold displacement $u_{y}$ and the stiffness ratio α, being instead its in-zoom view of a restricted area close to the origin on the left-hand side of the figure. The isolines indicate the magnitude of the prescribed displacement $\tilde{\Delta }$ [m]. The shaded region denotes the range of parameter combinations that support effective adhesion-based preserving geometric compatibility and serves as a design and optimization tool for robotic design.

Therefore, by defining the value of $u_{y}$ which replicates the effective friction arising on a given substrate, we can enter the design map (figure 11) and identify the feasible region for locomotion. Within this map, once $u_{y}$ is known, we can tune the stiffness ratio α accordingly to ensure that the inelastic trigger displacement $\tilde{\Delta }$ falls within the compatibility region. On the other hand, if a specific support is selected as the target environment for the engineered device, the stiffness ratio α can be determined from the contour plot in figure 11, once the displacement threshold $u_{y}$ is prescribed. This approach enables a substrate-informed design strategy, in which the mechanical and geometric parameters can be calibrated ad hoc to optimize the initiation and maintenance of stable and periodic locomotion on the chosen substrate, by appropriately defining the elastic properties of the material employed for device prototyping.

In addition, while our previous kinematic framework [38] effectively captures locomotion triggered by a prescribed inelastic displacement at the terminal proleg, from an engineering design perspective, it may also be crucial to estimate the energy cost of the physical actuator imposing this displacement (Δ) at the terminal proleg of the caterpillar-inspired soft robot. Neglecting the crawler's inertial and dissipative terms, the required input energy of the actuator, denoted as $E_{\text{input}}$, can be roughly estimated as the mechanical work done by the reaction force $R_{1}(\Delta )$ of the support at the terminal appendage of the system, as follows:

(4.1) $$E_{\text{input}}\propto \int_{0}^{\tilde{\Delta }}R_{1}(\Delta )\;d\Delta ,$$

where $R_{1}(\Delta )$ is de facto equal to the internal force of the first inter-mass spring between subunit 1 and 2 for the horizontal equilibrium condition (figure 1). Under linear elastic hypotheses, the input energy $E_{\text{input}}$ assumes the following form:

(4.2) $$E_{\text{input}}\propto \frac{1}{2}k_{1}{\left(u_{2}-\tilde{\Delta }\right)}^{2},$$

which can be better particularized by making explicit the analytical solution $u_{2}$ of the first mechanical configuration, provided in our past research [38]:

(4.3) $$E_{\text{input}}\propto \frac{1}{2}k_{1}{\left(\frac{\alpha^{4}+7\alpha^{3}+15\alpha^{2}+10\alpha +1}{2\alpha^{4}+13\alpha^{3}+25\alpha^{2}+14\alpha +1}-1\right)}^{2}{\tilde{\Delta }}^{2},$$

where $\tilde{\Delta }$ is a function of the stiffness ratio α and the adhesion threshold $u_{y}$, as already made explicit in expressions (2.3). Thus, the energetic cost of the external actuation of the prototyped system is strongly affected by both inter-masses and Winkler-like springs stiffness (i.e. $k_{1}$ and $k_{2}$) and the adhesion $u_{y}$, implicitly included in the term $\tilde{\Delta }$.

In order to demonstrate the potentials of the proposed framework from an engineering and prototyping design realm, a series of parametric analyses were performed, and the numerical results are presented and discussed below.

In figure 12, the results of a sensitivity analysis of the caterpillar-like locomotion behaviour have been reported, with respect to two key design parameters: the adhesion threshold $u_{y}$ (left-hand column: subfigures a– c) and the stiffness ratio α (right-hand column: subfigures d– f). Each plot shows the evolution of the displacements of each d.f. over all the substeps for one gait cycle. In the left-hand column (a–c), increasing the adhesion leads to a noticeable increment in the amplitude of the inelastic contraction $\tilde{\Delta }$, thus resulting in higher values of displacement at each subunit. In addition, the same trend can be registered by fixing the adhesion threshold and by increasing the stiffness ratios, with a higher difference in terms of magnitude among displacements of each d.f. In light of the aforementioned considerations, since the parameter $u_{y}$ incorporates the soft animal's mass—which increases effective friction and, consequently, adhesion—the effect of adding mass to the caterpillar-like soft robot is inherently captured within the current sensitivity analyses.

**Figure 12.**
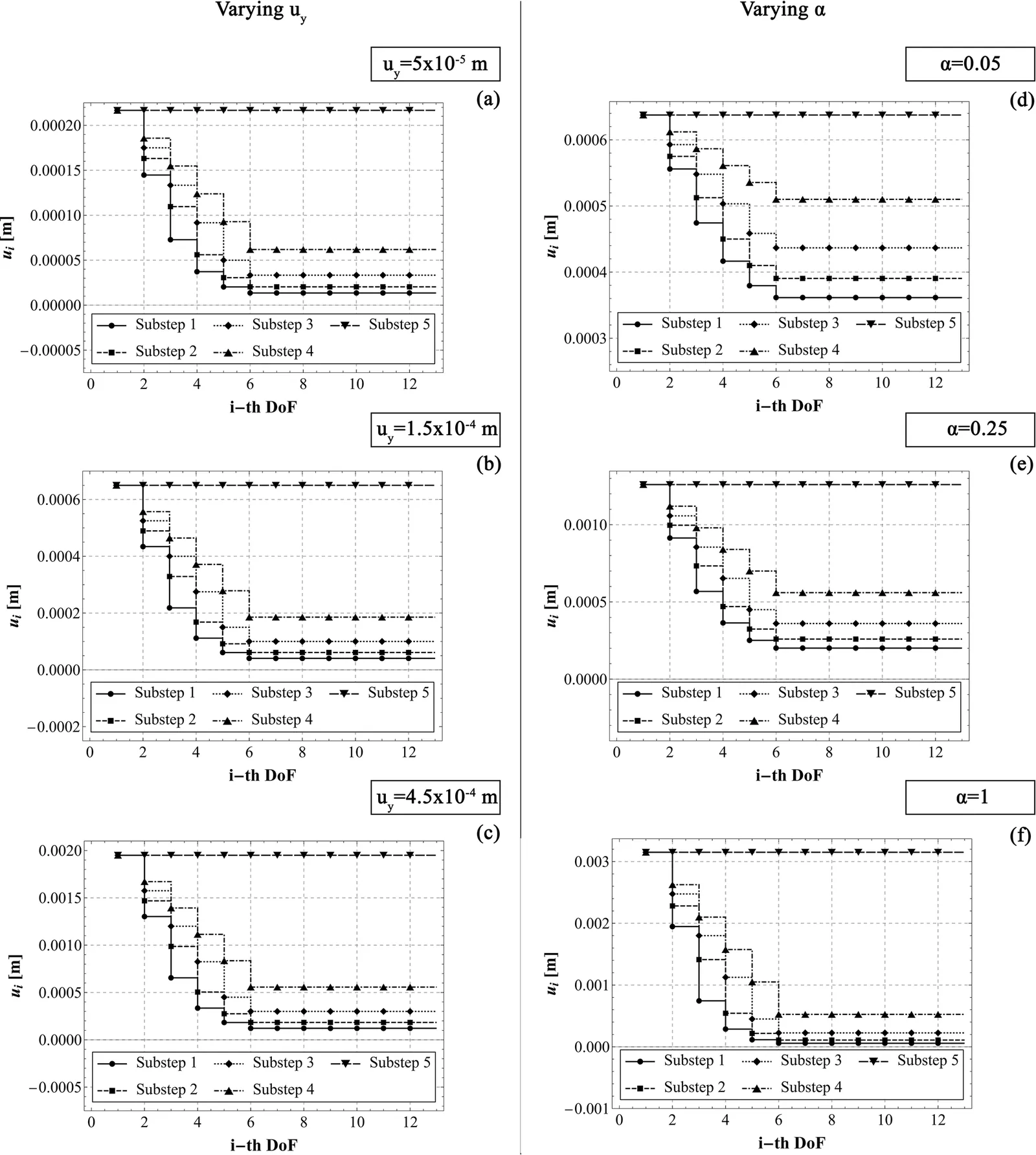
Displacements $u_{i}$ over the d.f. for the successive substeps of the locomotion cycle for selected values of displacement threshold $u_{y}$ (left-hand column: a–c) and stiffness ratio α (right-hand column: d–f). For all the numerical simulations provided here, the stiffness of inter-mass springs $k_{1}$ is set equal to 15 N m^–1^. The plots (a–c) reproduce the sensitivity of the displacement for each d.f. by keeping invariant $l_{0}=4\times 10^{-3}$ m and α=0.5 and by varying the displacement threshold $u_{y}$ from $5\times 10^{-5}$ to $4.5\times 10^{-4}$ m. Increasing $u_{y}$ corresponds to different substrate adhesion conditions and alters the inelastic contraction amplitude required to initiate locomotion (equation (2.3)). The plots (d–f) refer to the case where the adhesion threshold is fixed to $4.5\times 10^{-4}$ m, and the resting length of inter-mass springs $l_{0}$ is set to $4\times 10^{-3}$ m, while α varies from 0.05 to 0.5. Tuning α affects how deformations propagate across the structure, influencing anchoring and advancing movement for each substep.

Similarly, by taking advantage of the dynamic model analysed in [38] and briefly recalled in §2.1, the implications of the system's parameter variations are investigated with a particular focus on the transient regime. In figure 13, we compare the velocity histories of the second and twelfth d.f. at the first locomotion substep under variations of the displacement threshold $u_{y}$ (left-hand column) and the stiffness ratio α (right-hand column). In more detail, panels (a–c) highlight the role of adhesion sensitivity: as $u_{y}$ increases, the transient amplitudes of both d.f. are strongly affected, with higher thresholds leading to an increment of the maximum peaks and of the average response over the whole time duration.

**Figure 13.**
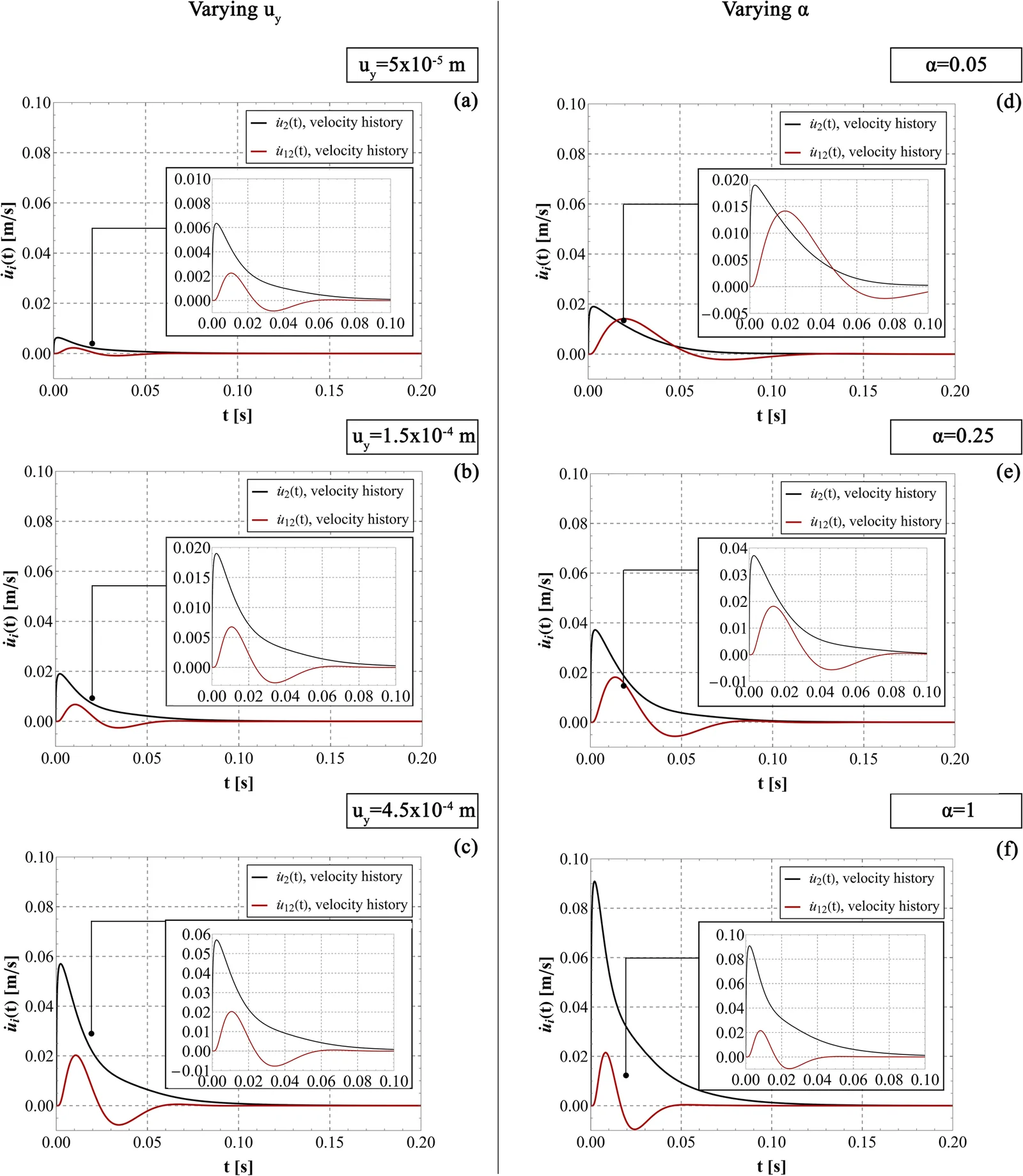
Velocity histories ${\dot{u}}_{i}(t)$ related to the first locomotion substep of the second and twelfth N m^–1^, while the subunit masses and the damping ratio are assumed, respectively, equal to $1\times 10^{-4}$ kg and 5, being instead $\tilde{\Delta }$ varying according to equation (2.3). Panels (a–c) illustrate the displacement sensitivity associated with two d.f., under constant values of $l_{0}=4\times 10^{-3}$ m and α=0.5, while the displacement threshold $u_{y}$ is varied within the range $5\times 10^{-5}$–$4.5\times 10^{-4}$ m. A higher value of $u_{y}$ corresponds to stronger substrate adhesion, which increases the transient velocities of both d.f. and results in greater kinetic energy accumulation during the initial stage. Panels (d–f) present the complementary case in which the adhesion threshold is fixed at $4.5\times 10^{-4}$ m, with the resting spring length set to $l_{0}=4\times 10^{-3}$ m, while α spans values from 0.05 to 0.5. Changes in α regulate and delay the propagation of deformations throughout the system, thereby influencing locomotion dynamics, and substantially altering the interdependence of the velocity histories of the d.f., with respect to the counterpart numerical analyses in the left-hand column.

By contrast, panels (d–f) show the influence of the stiffness distribution through the α parameter. For small values of α, the velocity histories are nearly similar in terms of maximum values, even if a delayed response is registered for the last d.f., somehow suggesting balanced energy propagation across the lumped mass chain modelling caterpillar locomotion. As α increases, the velocity profile of the second d.f. grows sharper and more pronounced, while the twelfth d.f. exhibits attenuated oscillations. This asymmetry demonstrates that increasing the stiffness ratio leads to a redistribution of the deformation energy along the body, favouring proximal d.f. and limiting distal propagation.

Therefore, the numerical results presented here underline the dual role of adhesion threshold and stiffness ratio: while $u_{y}$ predominantly governs the overall amplitude of the dynamic response, α modulates the spatial distribution of energy across the several d.f. even during the transient dynamic phase. In this light, the interplay between these two parameters affects the overall motion modalities of the soft-bodied animal.

## 5. Conclusions and future perspectives

In this work, we recovered and upgraded the discrete mechanical model of caterpillar locomotion developed in our previous studies, by further validating, both theoretically and experimentally, its capability to trace back locomotion outcomes by playing on the competition between segmental body elasticity and proleg adhesion, which is the main feature of the animal gait. While past research [38,51] analysed locomotion on wooden substrates—the familiar support on which caterpillars typically walk—the present paper aims to demonstrate how artificially altering the mechanical properties of caterpillars can modify their behaviour, allowing the soft-bodied animals to move on surfaces normally non-walkable for them.

With this in mind, we conducted an experimental campaign to test both real caterpillar motion and the capability of the model to capture locomotion on substrates by playing with different mechanical and adhesion properties, including surfaces not naturally encountered by the animal. To assess the robustness of the locomotion mechanism beyond its familiar support, we in particular conceived and designed ad hoc laboratory set-ups, where the characteristic times and velocities of the caterpillar gait on a wooden surface were numerically compared with those on other supports. Our observations showed that caterpillars, whose motion is designed by nature to be optimal on wooden substrates, adjust—quantitatively and qualitatively—gait features (e.g. velocity and cycles) as the material substrates change, the direction preferred by the animal to move depending on the eventual micro-patterns characterizing the support surface as well. In more detail, caterpillars belonging to the species *Pieris brassicae* reach approximately the velocity of 3.29 mm s^–1^ on the wooden support, registering a reduction of approximately 25% on the slate substrate, moving to 43–45% if compared to the steel bar and ultimately reaching a very low average velocity of 0.28 mm s^–1^ on glass surfaces, which correspond to a decrement of more than 90% with respect to the wooden material.

Importantly, we also proved that equipping caterpillars with articial stiffening armour and changing adhesion at the proleg-substrate interface through beds that influence effective friction can strongly modify locomotion performance. In particular, while the average velocity is penalized on its natural wooden support with a reduction of approximately 36.5%, the one measured on glass substrate increases to 570%, if one compares the crawling gait of caterpillars with or without artificial modifications.

By highlighting the synergistic interplay between adhesion and stiffness in caterpillar locomotion and by deciphering the main aspects related to the substrate dependence of caterpillars, these findings pave the way for a new generation of bioinspired soft robots that can be potentially designed for traversing any substrate.

## Data Availability

As mentioned in the main text of the manuscript, the codes and data employed for the research are based on the ones already provided by the authors in [73].
